# Glutamate Excitoxicity Is the Key Molecular Mechanism Which Is Influenced by Body Temperature during the Acute Phase of Brain Stroke

**DOI:** 10.1371/journal.pone.0044191

**Published:** 2012-08-28

**Authors:** Francisco Campos, María Pérez-Mato, Jesús Agulla, Miguel Blanco, David Barral, Ángeles Almeida, David Brea, Christian Waeber, José Castillo, Pedro Ramos-Cabrer

**Affiliations:** 1 Clinical Neurosciences Research Laboratory, Department of Neurology, Hospital Universitario de Santiago, University of Santiago de Compostela, IDIS, Santiago de Compostela, Spain; 2 Research Unit, Hospital Universitario de Salamanca and Institute of Health Sciences of Castilla and León, Salamanca, Spain; 3 Department of Biochemistry and Molecular Biology, University of Salamanca, Salamanca, Spain; 4 Department of Radiology, Massachusetts General Hospital, Charlestown, Massachusetts, United States of America; Massachusetts General Hospital/Harvard Medical School, United States of America

## Abstract

Glutamate excitotoxicity, metabolic rate and inflammatory response have been associated to the deleterious effects of temperature during the acute phase of stroke. So far, the association of temperature with these mechanisms has been studied individually. However, the simultaneous study of the influence of temperature on these mechanisms is necessary to clarify their contributions to temperature-mediated ischemic damage. We used non-invasive Magnetic Resonance Spectroscopy to simultaneously measure temperature, glutamate excitotoxicity and metabolic rate in the brain in animal models of ischemia. The immune response to ischemia was measured through molecular serum markers in peripheral blood. We submitted groups of animals to different experimental conditions (hypothermia at 33°C, normothermia at 37°C and hyperthermia at 39°C), and combined these conditions with pharmacological modulation of glutamate levels in the brain through systemic injections of glutamate and oxaloacetate. We show that pharmacological modulation of glutamate levels can neutralize the deleterious effects of hyperthermia and the beneficial effects of hypothermia, however the analysis of the inflammatory response and metabolic rate, demonstrated that their effects on ischemic damage are less critical than glutamate excitotoxity. We conclude that glutamate excitotoxicity is the key molecular mechanism which is influenced by body temperature during the acute phase of brain stroke.

## Introduction

Stroke is a leading cause of mortality and morbidity in developed countries, with increasing incidence due to the progressive aging of their population. Pharmacological or mechanical reperfusion therapy is the most effective treatment during the acute phase of ischemic stroke, and it is associated with good outcome in 50–70% of cases. However, these treatments are only applicable to less than 10% of patients, due to severe restrictions that include a short therapeutic window [1]. The management of body temperature is becoming one the most promising neuroprotective strategies during the acute phase of stroke, for those cases in which reperfusion is not recommended [2], [3]. Understanding the underlying mechanisms by which temperature affects the progression of ischemic tissue may lead to advances for the treatment of stroke.

Earlier studies have associated temperature effects with glutamate excitotoxicity and with alterations of the metabolic rate and the inflammatory response, as these processes were exacerbated by hyperthermia and reduced under hypothermic conditions [3]. So far, such associations have been studied in isolation, and we are not aware of studies in which the relevance of these three mechanisms was evaluated together.

Previously published studies [4], [5] prompted us to postulate that the most critical mechanism that affects temperature-related damage is glutamate excitotoxicity, compared with metabolic rate and inflammatory response. To test this hypothesis, we combined a pharmacological approach with temperature changes to alter brain glutamate levels. Our pharmacological approach is based on the fact that there is a direct correlation between glutamate levels in blood and in brain extracellular medium [6]. Thus, the increase of glutamate concentration in blood by systemic injection of this substance it is reflected in an increase of extracellular glutamate levels on the ischemic brain, exacerbating its excitotoxic effects. Conversely, reduction of blood glutamate concentrations by treatment with oxaloacetate, a co-substrate of the blood-borne enzyme glutamate oxaloacetate transaminase (GOT), leads to a reduction of extracellular glutamate in the brain, with a subsequent reduction of its excitotoxic effects (see [7] for review).

In the present work we have used non invasive techniques, such as Magnetic Resonance Spectroscopy (MRS) and Imaging (MRI), to study an animal model of ischemia under hypo-, normo- and hyperthermic conditions; we show that it is possible to modulate the deleterious effects of hyperthermia and the neuroprotective effects of hypothermia by pharmacologically influencing the excitotoxic effect of glutamate. The analysis of the effects of temperature on metabolic rate, inflammatory response and glutamate excitotoxicity indicates that the latter mechanism is the one that presents the strongest association with temperature.

**Figure 1:**
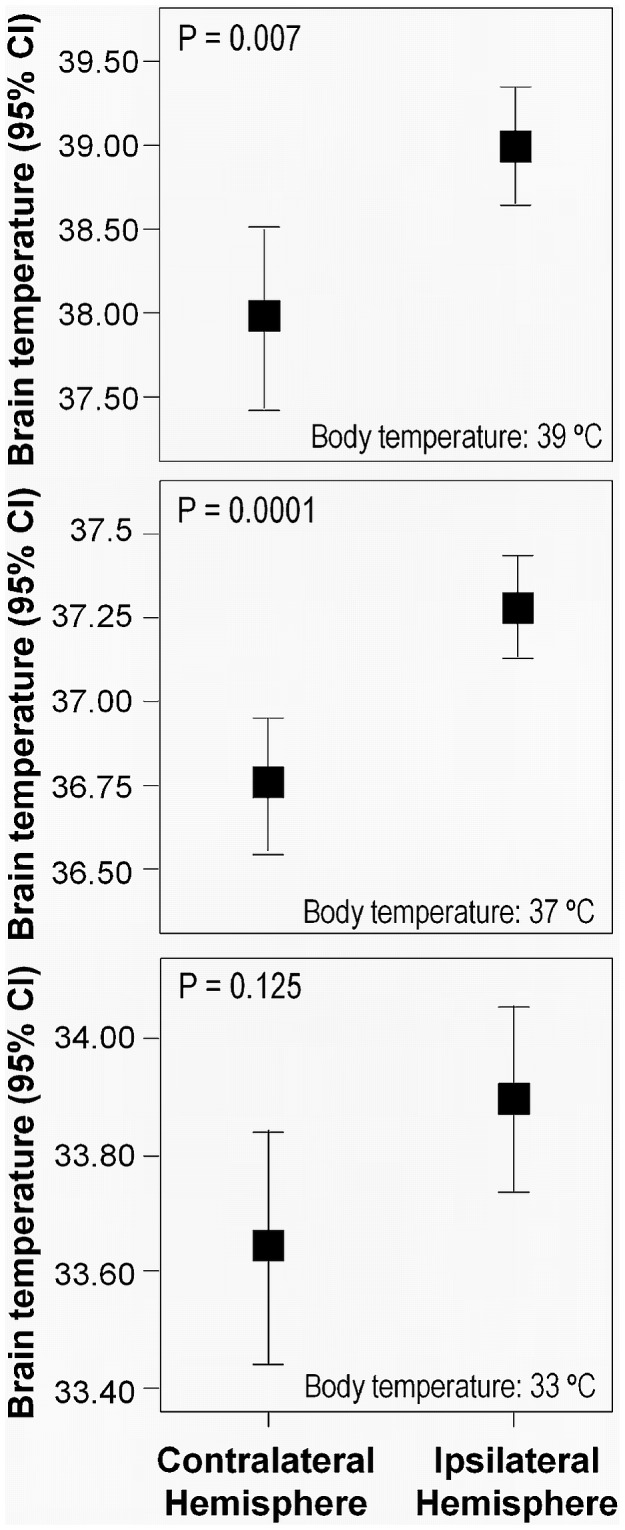
Brain temperature in the ischemic and the contralateral (healthy) brain hemispheres, as determined from Magnetic Resonance measurements on ischemic animals with body temperatures of 39°C (top), 37°C (middle) and 33°C (bottom). Statistical significances: *P<0.05.

## Materials and Methods

### Animals

Experimental protocols were approved by the local Animal Care Committee according to the Spanish and European Union legislation (86/609/CEE, 2003/65/CE, and RD 1201/2005). Male Sprague-Dawley rats (Harlan Laboratories, Udine, Italy) with a weight ranging from 300 to 330 g were used. Rats were provided with water and food *ad libitum*. Anesthesia was induced by inhalation of 5% sevoflurane in a nitrous oxide/oxygen mixture (70/30). Glucose levels remained at normal levels prior to surgery (180 to 220 mg/dl).

**Figure 2:**
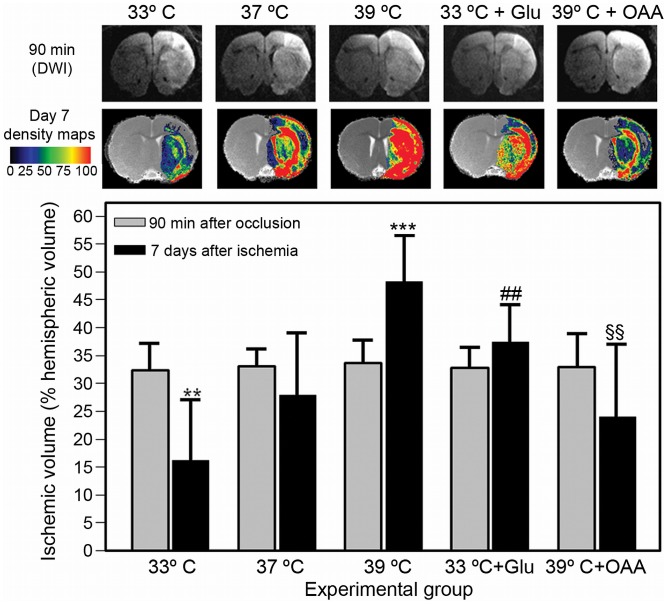
MRI images and quantification of infarct volumes at 90 min (DWI MR images) and 7 days (density maps from T2-MRI images) after the induction of cerebral ischemia in animals submitted to hypothermia (33°C, n = 8), normothermia (37°C, n = 7), hyperthermia (39°C, n = 7), hypothermia and 7,27 g/kg of glutamate (Glu) (n = 8) and hyperthermia (n = 8) and 35 mg/Kg oxaloacetate (OAA). ). Color-coded density maps were generated from T2 MR images from one brain slice for each animal (Located at Bregma +0.7 mm). The color code indicates the percentage of animals within a group that shows a lesion in each particular pixel. The grey image used as background is the result of the mean value of all co-registered images per group. Infarct volumes (% of hemispheric volume affected by lesion) are shown as mean ± S.D. Statistical significances: ***P<0.001, **P<0.01 compared to normothermic animals. ##P<0.01 compared to non-treated hypothermic animals, §§P<0.01 compared to non-treated hyperthermic animals.

### Surgical Procedures

Transient (90 min) focal ischemia was induced by intraluminal occlusion of the middle cerebral artery (MCA), as described elsewhere [8]. A laser-Doppler flow probe (tip diameter 1 mm) attached to a flow meter (PeriFlux 5000; PerimedAB, Stockholm, Sweden) was placed on the thinned skull over the MCA territory (4 mm lateral to Bregma) to continuously record the relative cerebral blood flow (CBF) during the experiment. Only animals with a reduction of CBF higher than 60%, and with effective reperfusion after retrieving the filament, were included in the study.

**Figure 3:**
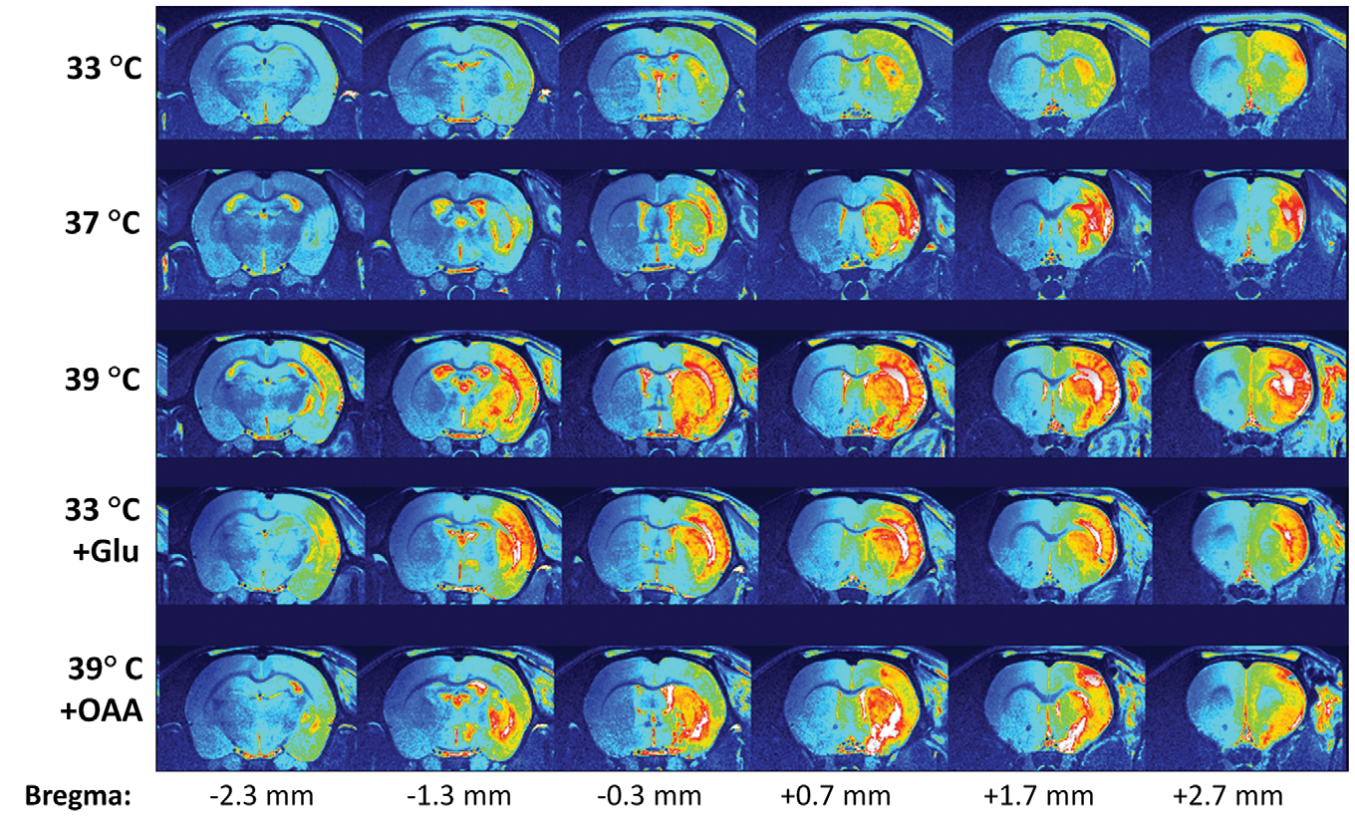
Pseudocolor T2 weighted MR images of a representative brain of each group. Images include 7 consecutive 1-mm thick coronal slices, showing the extent of the ischemic damage throughout the whole brain (position of each slice compared to Bregma, to Paxinos and Watson [45], is presented at the bottom).

### Experimental Groups

Two sets of experiments were designed:

1. Effect of temperature on glutamate levels on healthy rats - Three different groups of healthy animals were subjected to different body temperatures, hypothermia (33±1°C, n = 7), normothermia (37±1°C, n = 7) and hyperthermia (39±1°C, n = 7), to study the effects of temperature on blood glutamate concentration. Body temperature was maintained by using a feedback-controlled heating pad. Blood samples (200 µL) were withdrawn under basal conditions (normothermia) and after achieving the selected target temperatures.
2. Effect of temperature on ischemic rats - Five groups were used to study the effect of temperature and blood glutamate concentrations: group A: ischemic animals subjected to hypothermia (n = 8); group B: ischemic animals subjected to normothermia (n = 7); group C: ischemic animals subjected to hyperthermia (n = 7); group D: ischemic animals subjected to hypothermia and treated with 7.27 g/kg of glutamate (n = 8); group E: ischemic animals subjected to hyperthermia and treated with 35 mg/kg of oxaloacetate (n = 8). Glutamate and oxaloacetate were administered before the beginning of MCAo surgery. In each group, temperature (33°C, 37°C or 39°C ±1) was maintained from the beginning of the surgery (duration of surgery: 30–40 min), during MCA occlusion (90 min) and up to 150 min after reperfusion (duration of MR studies). For all groups, concentrations of blood glutamate and IL-6 were determined at the beginning of the experiment (basal values; before surgery and normothermia) and 90 min after occlusion of the MCA, just after reperfusion. For all groups, brain temperature and relative cerebral glutamate and lactate levels were determined by MRS during MCA occlusion and up to 150 min after reperfusion. To do so, animals were carefully taken from the surgery table after occlusion of the MCA and placed in the MRI system. An MR angiography was first performed to confirm that the MCA remained occluded. After 90 min of occlusion, animals were taken out of the MRI and the filament was removed. Subsequently animals were placed again in the MRI instrument and relative brain glutamate and lactate levels were analyzed by MRS for a period of 150 min after reperfusion. Infarct volumes were measured in all animals 90 min after the occlusion (by diffusion-weighted images) and 7 days later (T2-weighted images).

### Blood Glutamate Analysis

Blood samples were collected from the tail vein, centrifuged at 3000 g for 15 minutes and immediately frozen and stored at −80°C. Serum levels of glutamate were determined by high performance liquid chromatography (HPLC), as described elsewhere [9].

**Figure 4:**
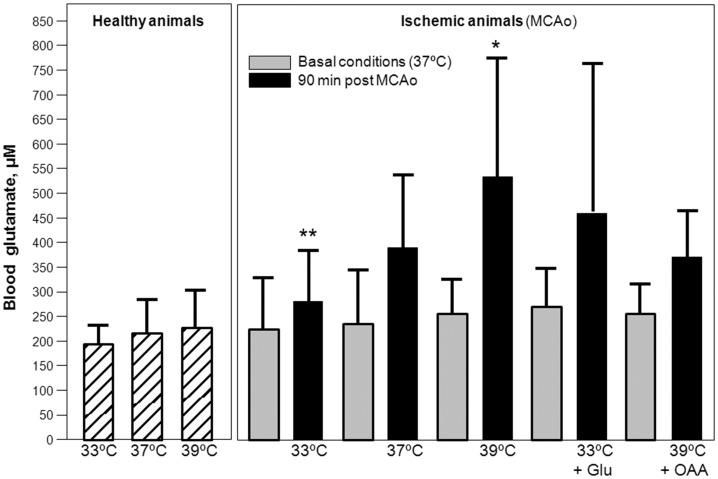
Blood glutamate levels (µM) in healthy and in ischemic animals submitted to hypothermia (33°C, n = 8), normothermia (37°C, n = 7), hyperthermia (39°C, n = 7), hypothermia plus 7.27 g/Kg glutamate (Glu) (n = 8) and hyperthermia plus 35 mg/Kg oxaloacetate (OAA) (n = 8) (A). Basal glutamate levels were determined in all groups under normothermic conditions. Ischemic levels were determined 90 min after occlusion. Levels are shown as mean±S.D. Statistical significances: **P<0.01, *P<0.05 compared to normothermic animals. Brain glutamate levels (corrected as the ratio between glutamate and creatine MRS peak) for the same groups of animals (**B**). Glutamate levels were determined during the occlusion (90 min) and after reperfusion (180 min). Levels are shown as mean±S.D. ***P<0.001, *P<0.05 compared to normothermic animals.

### Plasma IL-6 Analysis

Plasma levels of IL-6 were determined using an immunodiagnostic IMMULITE 1000 System (Diagnostic Products Corporation, California, USA). Levels of IL-6 were analyzed under basal conditions (37°C, before surgery), and 90 min after occlusion of the MCA, just after reperfusion.

**Figure 5:**
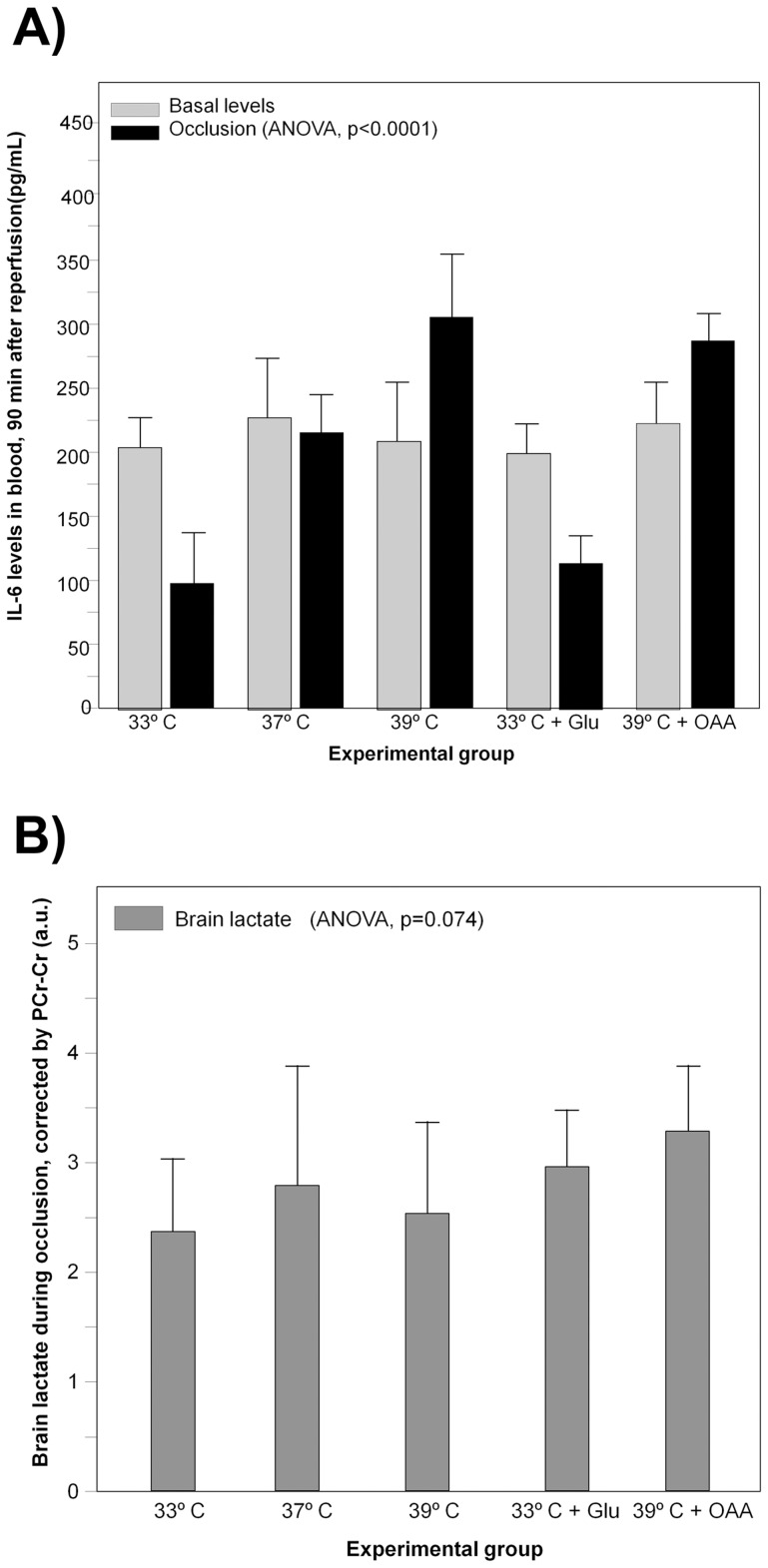
Blood Interleukin-6 levels (in pg/mL) in animals submitted to hypothermia (33°C, n = 8), normothermia (37°C, n = 7), hyperthermia (39°C, n = 7), hypothermia plus 7.27 g/Kg glutamate (Glu) (n = 8) and hyperthermia plus 35 mg/Kg oxaloacetate (OAA) (n = 8) (A). Lactate levels during occlusion period (expressed as the ration glutamate/creatine-phosphocreatine peak areas on MRS spectra) on the ischemic brain of the same group of animals (**B**).

### Magnetic Resonance Imaging: Infarct Volume Analysis

MRI and MRS studies were conducted on a 9.4T horizontal bore magnet (Bruker BioSpin, Ettligen, Germany) with 20 cm wide actively shielded gradient coils (440 mT/m). Radio frequency transmission was achieved with a bird cage volume resonator; signal was detected using a four elements surface coil positioned over the head of the animals. Infarct sizes were assessed from MRI images. Apparent diffusion coefficient (ADC) maps were acquired before removing the filament from the animal (90 minutes after the occlusion) using a spin-echo echo-planar imaging sequence with the following acquisition parameters: field-of-view 19.2×19.2 mm^2^, image matrix 128×128 (in-plane resolution 0.15 mm/pixel), 14 consecutive slices of 1 mm thickness, repetition time  = 4 s, echo time  = 30 ms, and diffusion b values: 0, 100, 300, 600, 800, 1000, and 1400 s/mm^2^. T2-weighted images were acquired 7 days after the induction of ischemia using a multi-slice multi-spin-echo sequence with the following acquisition parameters: field-of-view 19.2×19.2 mm^2^, image matrix 192×192 (isotropic in-plane resolution of 0.1 mm/pixel), 14 consecutive slices of 1 mm thickness, repetition time  = 3 s, and 16 echoes with echo time  = 9 ms.

### In vivo Magnetic Resonance Spectroscopy: Glutamate and Lactate Analysis

MR spectra were acquired as described elsewhere [10], [11]. Localized spectroscopic data were collected from a 3×3×3 mm^3^ voxel (27 µL) covering part of the cortex and striatum of the stroke-affected hemisphere (mostly filled by ischemic tissue). A second voxel was selected at the contralateral hemisphere (healthy tissue), for reference. Local shimming was performed by manual adjustment of first- and second-order shim coils, using a proton stimulated-echo acquisition mode (STEAM) pulse sequence. Field homogeneity resulted in typical signal line widths of 10–20 Hz for the water peak. Water signal was suppressed using a VAPOR scheme (bandwidth  = 350 Hz). In vivo 1H spectra of the rat brain were acquired by using a STEAM sequence with echo time TE = 3 ms, mixing time TM = 5 ms, repetition time TR = 2.5 s, N = 176 averages. Outer volume suppression was applied. Spectra were processed using TOPSPIN 2.0 (Bruker Biospin, Ettlingen, Germany) and M-Nova (MestreLab Research, Santiago de Compostela, Spain). For the quantitative analysis, glutamate and lactate signals were normalized with the creatine peak areas, used as internal reference, for each single spectrum.

### Brain Temperature Estimation

Mapping of brain temperature by MR has been previously validated [12], [13]. Cerebral temperature for each voxel was calculated based on the fact that temperature-dependent changes in hydrogen bonding linearly affect the chemical shift of water (δ_H2O_), with a slope of 0.01 ppm per °C [14], whilst the chemical shift of N-acetyl aspartate (δ_NAA_) is basically independent of temperature.

We validated the MRS thermometry technique in our 9.4 T system with a 1 mM solution of N-acetyl aspartate (NAA) subjected at a range of temperatures from 30 to 40°C. Statistical analysis showed a Pearson’s Coefficient = 0.965 (p<0.0001) between temperature and the difference of chemical shifts of water and N-acetyl aspartate (Δ = δ_H2O_−δ_NAA_). To determine the relationship between chemical shift differences and brain temperature, we selected two groups of healthy animals (n = 9 each), inserting a temperature micro-sensor in the brain of the animals of one of the groups, and measuring the temperature of the brain inside the magnet, while body temperature was subjected to a range of temperatures from 30 to 39°C. A second group of animals was placed in the MR and subjected to the same range of body temperatures, measuring the chemical shifts of NAA and water. Pearsońs correlation coefficients were 0.943 (p<0.0001) and 0.893 (p<0.0001), respectively. Based on this analysis, we were able to derive the following equation to estimate brain temperature from MRS measurements: cerebral temperature (Ct) = 237.279−(75.289×Δ), where Δ reflects the difference of values of the chemical shift of NAA and water (in PPM).

### Image Analysis

All images were processed with ImageJ (Rasband WS, ImageJ, US National Institutes of Health, Bethesda, MD, USA, http://rsb.info.nih.gov/ij/, 1997–2009). Infarct volumes were determined from quantitative ADC (apparent diffusion coefficient) and T2 maps by a researcher blinded to animal treatment.

### Statistical Analysis

Results are expressed as percentages for categorical variables and as mean (± standard deviation) or median (quartiles) for continuous variables, depending on the normality of the data distribution. Proportions were compared using the χ^2^ test. The Student’s t-test or the Mann–Whitney test were used to compare continuous variables between groups. Spearman or Pearson analyses were used for bivariate correlations. ANOVA test was used to compare multiple quantitative variables.

## Results

### Brain Temperature in the Ischemic Brain

Brain temperature was determined in ischemic animals by non-invasive MRS measurements, under hypo-, normo- and hyperthermic conditions (Figure 1). Reducing body temperature to 33°C was paralleled by a reduction in brain temperature, with no significant differences between ipsilesional and contralateral brain hemispheres (p = 0.125). In hyperthermic animals, an increase of body temperature to 39°C was reflected in an increase of brain temperature. At variance with hypothermic animals, under normo- and hyperthermia conditions significant temperature differences (p = 0.0001 and p = 0.007, respectively) were observed between the ipsilesional and the contralateral brain hemispheres. In general, changes in body temperature were associated with changes in the temperature of the ischemic brain, with higher temperatures (differences up to 0.5°C) observed for the ipsilesional brain hemisphere compared to the contralateral (healthy) side.

### Effects of Body Temperature on the Ischemic Damage

Infarct volumes were measured in ischemic animals submitted to different experimental conditions at 90 min (diffusion-weighted images) and 7 days (T2-weighted images) from the induction of ischemia (Figure 2). Infarct volumes (expressed as % of the hemispheric volume affected by ischemia) were not different for all the studied groups at 90 min from the occlusion of the MCA (p = 0.995).

Under hypothermic conditions, infarct volumes were reduced at 7 days after occlusion (V = 15±10.8% of hemispheric volume) compared to their initial value at 90 min (V = 32±4.0% of hemispheric volume) (p<0.0001). Such reduction in lesion volumes was not detected for normothermic animals (V = 32±3.4% at 90 min vs. 27±11.9% at day 7) (p = 0.079). Conversely, infarct volumes were increased in animals subjected to hyperthermia (V = 33±3.2% at 90 min and V = 47±8.0% at day 7) (p<0.0001). Infarct volumes at 7 days were significantly smaller for hypothermic animals (p = 0.003), and larger for hyperthermic animals (p<0.0001), with compared to normothermic animals (Figure 2).

The reduction in infarct volumes induced by hypothermia was not seen in animals pretreated with glutamate, which showed a lesion volume of V = 36±7.4% at day 7 compared to an initial value of V = 32±3.2% at 90 min (p = 0.308). Similarly, the increase in lesion volumes observed for hyperthermic animals was not seen after treatment with oxaloacetate (V = 24±11.9% at day 7, vs V = 32±7.4% at 90 min) (p = 0.065).

The extent of ischemic damage along the whole brain for the different study groups is shown in Figure 3. Hyperthermia, as well the injection of glutamate, extended the damage toward the caudal sections of the brain. Conversely, hyperthermia and treatment with oxaloacetate reduced the intensity of the damage on the tissue.

### Effect of Temperature on Blood and Brain Levels of Glutamate under Ischemia

Under normothermic conditions (Figure 4A), 90 minutes of ischemia induced an increase in blood glutamate levels from the basal value of [glutamate]_basal_  = 237±111.9 µM to [glutamate]_90 min_ (37°C) = 380±167.4 µM, (p = 0.018). Such difference was smaller under hypothermic conditions ([glutamate]_basal_  = 229±97.9 µM vs [glutamate]_90min_ (33°C) = 278±88.1 µM, p = 0.028), and larger under hyperthermic conditions ([glutamate]_basal_ = 251±75.1 µM vs [glutamate]_90 min_ (39°C) = 530±233.1 µM, p = 0.016). Systemic treatment with 7.27 g/kg glutamate in hypothermic animals increased blood glutamate levels during occlusion ([glutamate]_90 min_ (33°C+glutamate) = 411.1±302.1 µM). These values were similar to those obtained for normothermic animals (p = 0.157). In hyperthermic animals, oxaloacetate treatment (35 mg/kg) reduced blood glutamate levels, ([glutamate]_90 min_ (39°C+oxaloacetate) = 376.6±104.8 µM) to levels seen in normothermic animals (p = 0.159) (Figure 4A). Basal levels, measured in normothermic conditions and before any treatment, were not different among all studied groups (p = 0.950). Body temperature did not affect blood glutamate levels in healthy rats (p = 0.175) (Figure 4A).

In the ischemic brain, hypothermia caused a significant reduction of brain glutamate levels during the occlusion (Glu/Cr-PCr)_OCL_ (33°C) = 1.9±0.5 and reperfusion (Glu/Cr-PCr)_REP_ (33°C) = 0.8±0.4, compared with normothermic animals (Glu/Cr-PCr)_OCL_ (37°C) = 2.6±1.0 and Glu/Cr-PCr)_REP_ (37°C) = 1.6±0.8 (p<0.0001 and p<0.0001 respectively) (Figure 4B). Conversely, hyperthermia induced a significant increase in glutamate levels during occlusion Glu/Cr-PCr)_OCL_ (39°C) = 3.2±1.6) and reperfusion (Glu/Cr-PCr)_REP_ (39°C) = 2.4±1.3, compared to normothermic animals (p = 0.023 and p = 0.029 respectively) (Figure 4B). The effect of hypothermia and hyperthermia on brain glutamate was prevented when animals were systemically treated with glutamate or oxaloacetate, respectively. Systemic treatment with 7.27 g/kg glutamate in hypothermic animals increased brain glutamate levels during occlusion (Glu/Cr-PCr)_OCL_ (33°C+GLU) = 2.5±0.6, and reperfusion (Glu/Cr-PCr)_REP_ (33°C+GLU) = 1.4±0.4. These values were similar to those obtained for normothermic animals (p = 0.427 and p = 0.109, respectively). In hyperthermic animals, oxaloacetate treatment (35 mg/kg) reduced brain glutamate levels, (Glu/Cr-PCr)_OCL_ (39°C+OAA) = 2.2±1.1 and (Glu/Cr-PCr)_REP_ (39°C+OAA) = 1.8±1.1, to levels seen in normothermic animals (p = 0.079 and p = 0.107, respectively) (Figure 4B). Glutamate levels in contralateral region (non ischemic region) were used as control region. Both ischemia, temperature, glutamate or oxaloacetate treatments did not modified glutamate levels in this region.

### Effect of Temperature on Acute Inflammatory Response under Ischemia

Levels of Interleukin 6 (IL-6) were determined under basal conditions and 90 min after induction of ischemia, under the five study conditions (Figure 5A). No significant differences were observed for basal concentrations (global mean  = 219±31.15 pg/mL, p = 0.640). This value remained at the same levels after 90 minutes of ischemia for normothermic animals (215±30.23 pg/mL) (p = 0.463), while it was reduced under hypothermic conditions (99±27.90 pg/mL) (p<0.0001), and increased under hyperthermia (304±51.03 pg/mL) (p<0.0001).

Pharmacological modulation of systemic levels of glutamate did not affect to IL-6 levels. Hypothermic animals treated with glutamate (109±20,62 pg/mL) or hyperthermic animals treated with oxaloacetate (286±20.11 pg/mL) showed values similar to those observed at the same temperatures, without pharmacological treatment (p = 0.107 and p = 0.377, respectively) (Figure 5A).

### Effect of Temperature on Brain Metabolic Rate under Ischemia

Brain levels of lactate, as determined by MRS were found to be higher during (following) occlusion and were not significantly different (p = 0.074) among all studied groups; LAc/Cr-PCr ratio  = 2.45±0.62 (33°C), 2.73±0.88 (37°C), 2.48±0.85 (39°C), 2.78±0.06 (33°C+Glu) and 2.28±0.65 (39°C+OAA) (Figure 5B).

## Discussion

The MRI data presented in this study show that animals submitted to hyperthermia present with larger lesion volumes at day 7, compared to normothermic animals and hypothermic animals, which show the smallest lesion volumes (Figure 2 and 3). The fact that hyperthermia exacerbates ischemic damage while hypothermia offers a neuroprotective effect during acute ischemia is not new, and the role of temperature in cerebral ischemia has been extensively discussed in the literature (see [3] and [15] for recent reviews on this topic).

The novelty of this work relies on the use of pharmacological approaches to modulate glutamate levels on the brain, which has enabled us to demonstrate the tight association existing between temperature and glutamate excitotoxicity, and evaluate the mechanisms of action underling the effects of temperature during acute phase of stroke.

The successful combination of hypothermia with pharmacological strategies, based on the reduction of glutamate excitotoxicity, has been already proposed [16], [17], but none of these works really demonstrate an association between both mechanisms. Here we have followed a different approach, as we modulated the brain levels of glutamate by acting on systemic levels, in order to evaluate by non-invasive means the role of glutamate release on the effects of temperature. Our findings indicate that systemic administration of glutamate leads to an increase of brain glutamate levels in the infarcted regions (though not in the contralateral region), and reverses the neuroprotective effects of hypothermia. Moreover, systemic administration of oxaloacetate reduces brain levels of glutamate and prevents the deleterious effects of hyperthermia, reducing lesion volumes at day 7 to values observed for untreated normothermic animals (Figure 2). Therefore, our data suggest that temperature effects during acute ischemia are strongly associated to glutamate excitotoxicity.

It is well known that during cerebral ischemia, glutamate excitotoxicity occurs when a large amount of glutamate is released from brain tissue into the extracellular space, as documented by *in vitro* studies [18], *in vivo* animal studies [19], [20], and in ischemic patients [5], [21], [22]. However the mechanism throughout temperature modifies the brain glutamate release is not completely understood. Cerebral ischemia results from a reduction of complete loss of cerebral blood flow, followed by a depletion of ATP and occurrence of anoxic depolarization and spreading depression. These molecular events lead to a large increase of glutamate from intracellular space to extracellular space; which stimulates NMDA receptors and leads to increased intracellular calcium levels [23]. Based on this sequence of molecular process, it is tempting to speculate that the effect of temperature on glutamate release is mediated through its effects on ATP depletion and the occurrence of anoxic depolarization. However, in our point of view, temperature induces a pleiotropic effect; there is not a unique molecular mechanism able to explain the effect of temperature on glutamate release.

In agreement with previous clinical studies [5], [22], we have observed that hypothermia induces a reduction of blood glutamate levels with respect to normothermic animals, and hyperthermia induces the opposite effect; a pattern similar to the one observed in brain glutamate levels. The most acceptable explanation of this effect is that ischemia causes a permeability and/or disruption of the brain blood barrier (BBB) in the infarct region [24], [25], [26], allowing the diffusion of glutamate from brain to blood. However it is possible that there could be another mechanism by which body temperature affects blood glutamate homeostasis and its levels. Based on this idea and since alterations of blood glutamate can influence brain glutamate levels [6], [27], [28], [29], [30], [31], we wanted to prove the influence of temperature on blood glutamate.

With this purpose, healthy animals were submitted to hypo-, normo- and hyperthermia. We observed no difference in blood glutamate levels among the three groups. Thus, our data suggest that temperature directly affects brain levels of glutamate rather than glutamate homeostasis in the body, supporting the idea that blood glutamate concentration is one of the best markers of neuronal damage in stroke patients [32].

To test to what extent temperature has an effect on the metabolic rate under ischemia; we measured brain levels of lactate by MR spectroscopy. Lactate is a marker of anaerobic metabolism and is thought to be correlated with the level of oxygen deprivation and ischemia [33], [34], [35]. We hypothesized that lactate levels should be temperature dependent on the ischemic brain if temperature affects metabolic rate. Our study shows no association between lactate levels and brain temperature or infarct volumes for any of the studied groups.

Other authors have related elevated temperatures with higher metabolic rates [36], [37]. However, a comparison of patterns of lactate concentration and temperature elevation in different regions of the ischemic brain showed that there was no causal association between both parameters [13]. The most plausible explanation for the correlations between metabolic rate and temperature relies on the fact that brain temperature is mainly elevated in the ischemic penumbra, an area of elevated metabolic rate [12]. The higher temperature of the ischemic penumbra may be the consequence of alterations of cerebral blood flow and an inflammatory response, which takes place locally at this ischemic region during early stages of ischemic stroke. Reduced blood flow could impair heat exchange resulting in higher temperatures at the ischemic lesion, where cells may still be metabolically active.

Similarly, the local inflammation response is linked to the elevation of tissue temperature, which appears minutes after induction of ischemia [2], [3]. It is well established that inflammation plays a key role in the physiopathology of ischemic stroke [38], [39]. The existence of an inflammatory cascade with pyretic activity that is associated with the activation and infiltration of microglia, macrophages and leukocytes (especially at the peri-infarct zone) has been demonstrated in animal models [40]. The beneficial effects of hypothermia have been related to the inhibition of the cellular response (accumulation of inflammatory cells in the peri-infarct zone) and of the humoral response (release of pro-inflammatory cytokines) to stroke [41].

Blood levels of interleukin 6 (IL-6), a known marker of early inflammation [41], [42], [43], show that the immune response is inhibited by hypothermia and exacerbated by hyperthermia. However, the fact that in our study levels of IL-6 were not affected by the pharmacological modulation of glutamate levels, as was temperature, may indicate a non causal relationship between temperature and the inflammatory response. Such relationship may not be the same in later phases of stroke. We have recently demonstrated in a clinical study that the therapeutic window for hypothermia persists for at least 48 hours, and that it is associated with inflammatory mechanisms [2]. Experimental studies have also demonstrated that hypothermia induces neuroprotective effects even when this treatment is delayed by up to 6 h [2], [3], [44]. It is the possible that the inflammatory response may be mainly associated with the neuroprotective effect of hypothermia in later stages of ischemia.

In conclusion, the present study shows that, among the different mechanisms of damage during the acute phase of stroke, excitotoxicity of glutamate is the most closely related to the effects of temperature during this stage. Causal relationships between temperature and other mechanisms of damage, like the presence of increased metabolic rates or inflammatory response, may become more relevant during the subacute or chronic phases of the disease. Based on our findings, we postulate that neuroprotective strategies inhibiting glutamate excitotoxicity may enhance the beneficial effects of hypothermia during the acute phase of stroke.
